# Analysis of aqueous humor total antioxidant capacity and its correlation with corneal endothelial health

**DOI:** 10.1002/btm2.10199

**Published:** 2020-12-05

**Authors:** Yu‐Ting Tsao, Wei‐Chi Wu, Kuan‐Jen Chen, Lung‐Kun Yeh, Yih‐Shiou Hwang, Yi‐Jen Hsueh, Hung‐Chi Chen, Chao‐Min Cheng

**Affiliations:** ^1^ Institute of Biomedical Engineering, National Tsing Hua University Hsinchu Taiwan; ^2^ Department of Education Chang Gung Memorial Hospital Linkou Taiwan; ^3^ Department of Ophthalmology Chang Gung Memorial Hospital Linkou Taiwan; ^4^ Department of Medicine Chang Gung University College of Medicine Taoyuan Taiwan; ^5^ Center for Tissue Engineering Chang Gung Memorial Hospital Linkou Taiwan; ^6^ Institute of Biomedical Engineering and Frontier Research Center on Fundamental and Applied Sciences of Matters, National Tsing Hua University Hsinchu Taiwan

**Keywords:** aqueous humor, ascorbic acid, cornea, corneal endothelial cell density (ECD), proteomics, total antioxidant capacity (TAC)

## Abstract

Corneal endothelial decompensation is a serious condition that frequently requires treatment via corneal transplantation which contributes to a global shortage in donor corneas. Therefore, the purpose of this study was to analyze the influence of aqueous humor total antioxidant capacity (TAC) on corneal endothelial health. There is an urgent need for discovering protective factors to combat corneal endothelial cell (CEC) loss. For methods, we developed a cupric ion‐based TAC (CuTAC) assay to analyze TAC level in a small volume of aqueous humor, that is, 10 μL per test, and examined the influences of ascorbic acid (AA) and antioxidant proteins on aqueous humor TAC. To broaden the investigation, we conducted a case–control study with patients classified into two groups, an insufficient endothelial cell density (ECD < 2100 cells/mm^2^) group, and a control group. These groups were formed based on baseline ECD values and were used to evaluate the influence of aqueous humor TAC and AA on overall corneal endothelial health. A CuTAC assay was used to accurately measure aqueous humor TAC without the need for sample dilution. After analyzing a total of 164 human aqueous humor samples, we found that AA was the major contributor to aqueous humor TAC (73.2%). In addition, TAC and AA levels in the IECD and control groups were both found to be significantly different (1.168 vs. 1.592 mM, *p* = 0.009 and 0.856 vs. 1.178 mM, *p* = 0.016). TAC and AA were considered independent protective factors against IECD with adjusted odds ratios of 0.02 (*p* = 0.017) and 0.023 (*p* = 0.033), respectively. In conclusion, aqueous humor TAC and AA contribute to the maintenance of sufficient corneal ECD, and our CuTAC assay can be a useful tool for analyzing TAC using only a small aqueous humor sample volume.

## 1 INTRODUCTION

Globally, corneal disease is the fourth leading cause of blindness. Approximately 5 million people suffer from severe vision impairment due to corneal opacity worldwide.^1^ The transparency of the human cornea relies on precise water regulation within and among the corneal endothelial cells (CEC). However, despite their importance in maintaining corneal function, human CEC are fragile and nonproliferative in vivo. Thus, corneal transplantation remains the primary treatment method for severe corneal injuries, which leads to a global shortage in donor corneas. There is an urgent need for discovering protective factors to combat CEC loss. Clinically, endothelial cell density (ECD) is one of the most important parameters for evaluating corneal health.^2^ ECD in infants is approximately 3500–4000 cells/mm^2^, a density that gradually decreases over time, and may drop significantly when injured.^3^, ^4^ An ECD value of 2100 cells/mm^2^ is thought to be the minimum standard for phakic intraocular lens implantation and cornea donation.^5^, ^6^, ^7^ A baseline ECD of less than 2100 cells/mm^2^ is further considered a marked risk factor for iatrogenic endothelial decompensation, which presents as a decline in ECD to levels less than 1000 cells/mm^2^.^8^ From this, we can deduce that patients with insufficient ECD (IECD, ECD less than 2100 cells/mm^2^) are poor candidates for intraocular surgeries. In addition, recognizing the pathophysiology behind IECD could potentially prevent and control undesirable ECD decline.

Although the exact mechanisms for CEC loss are still vague, reports have noted that free‐radical‐induced oxidative stress could be a chief cause.^9^ Human eyes are constantly subjected to ultraviolet light‐induced oxidative stress, and the anterior portions of the eye are responsible for absorbing ultraviolet radiation and protecting the posterior photoreceptor cells.^10^ However, once the oxidative stress overwhelms the antioxidant capacity of CEC, damage may occur. Diseases such as glaucoma, diabetic retinopathy, infectious ocular diseases, shallow anterior chamber, branch retinal vein occlusion, pterygium, and Sjogren's disease may all lead to increased oxidative stress and subsequent dysfunction among CEC.^11^, ^12^, ^13^, ^14^, ^15^, ^16^ It should also be noted that ocular surgery, especially cataract surgery, is an important risk factor for CEC loss and subsequent corneal blindness.^17^ For this reason, preoperative ECD evaluation is important and recommended. On the other hand, it has recently been reported that taking antioxidant supplements before or during the phacoemulsification process of cataract surgery can protect CEC from free radical damage.^18^, ^19^ In addition, several in vitro studies have also shown that antioxidants can promote CEC proliferation and prevent apoptosis.^20^, ^21^, ^22^ Despite this body of knowledge, there have not been any clinical studies directly showing the relationship between ECD and aqueous humor antioxidants.

**FIGURE 1:**
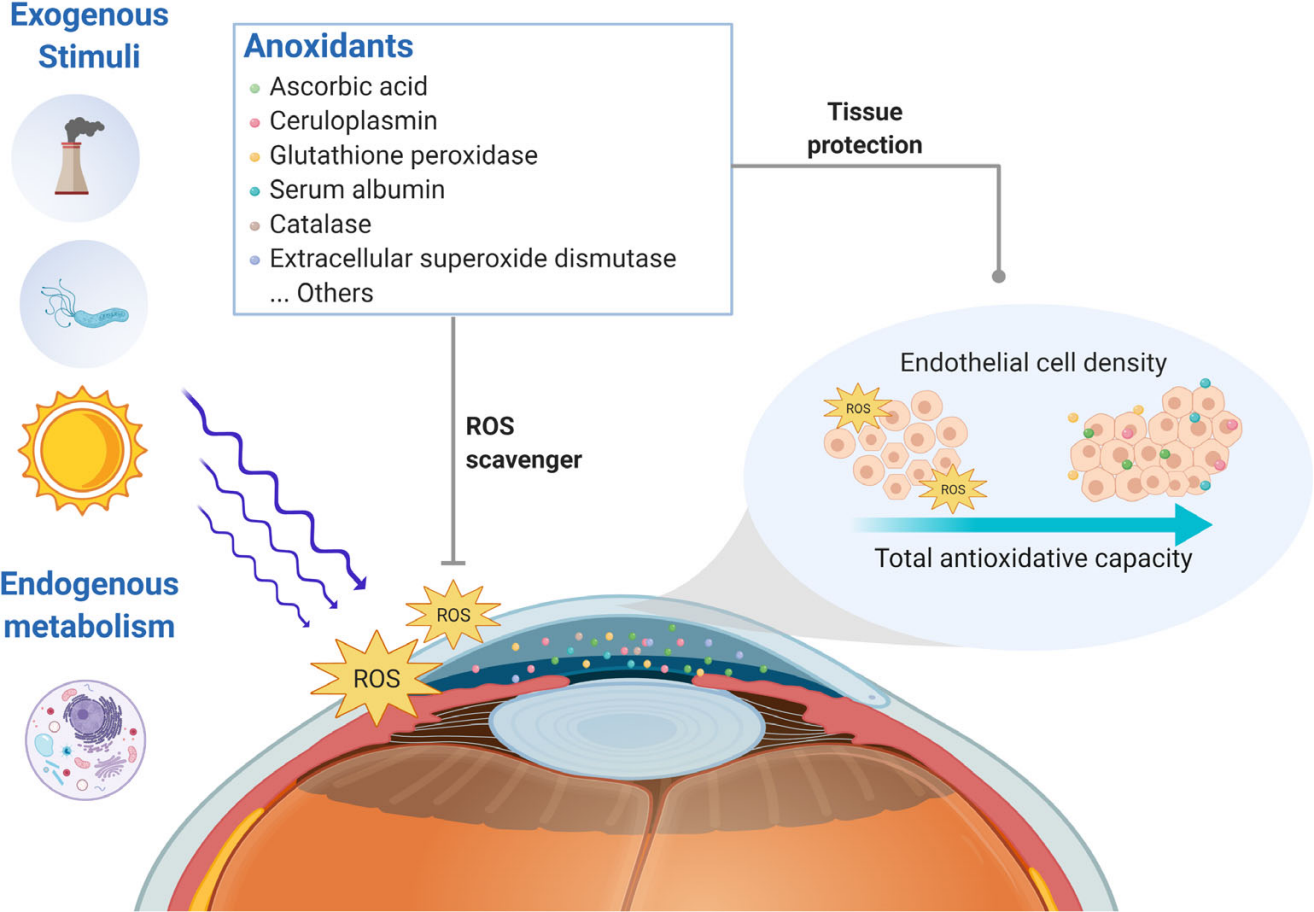
Schematic diagram illustrating the hypothetical role of the aqueous humor antioxidant defense system in protecting against the corneal endothelial cell (CEC) loss. Ocular tissues are subjected continuously to both exogenous and endogenous stimuli, including pollutions, microbial invasions, physical metabolism, and, most importantly, ultraviolet radiation. These stimuli give rise to toxic oxidant species and cause cell damage. However, because human CECs are fragile and nonproliferative in vivo, the antioxidants in aqueous humor that directly contact with the corneal endothelium should take responsibility for quickly eliminating free radicals and maintaining the integrity of the cells, and further prevent endothelial cell density loss. (The figure is created using BioRender.com)

Unlike most human tissues, corneal endothelium is deprived of blood vessels and nourished mainly by aqueous humor, which fills the anterior and posterior chambers of the eye and provides nutrients and antioxidants to intraocular tissues along its drainage path. Because the corneal endothelium is in direct contact with aqueous humor, we hypothesized that aqueous humor total antioxidant capacity (TAC) influenced ECD value, and further affected corneal endothelial health. Figure 1 illustrates the potential mechanism model by which aqueous humor TAC protects CEC from damage and ECD reduction. To protect the fragile and nonproliferative CEC from free radicals generated from exogenous stimuli or endogenous metabolism, the antioxidants in aqueous humor should quickly eliminate the free radicals and restore cell integrity. Therefore, aqueous humor TAC may be a key factor in determining CEC health and maintaining ECD.

It is difficult to develop aqueous humor analyses because of the small sample volumes available. Only about 200 μL of aqueous humor can be aspirated at a time before risking anterior chamber collapse. For this reason, there are no clinically available biochemical analyses based on aqueous humor, and thus no corresponding treatment strategies. For this reason, we developed a small‐sample‐volume TAC assay to measure aqueous humor TAC. As a means of further understanding the actual components of aqueous humor TAC, we measured the concentration of ascorbic acid (AA) and analyzed the antioxidant proteomics in aqueous humor. In addition, we have examined aqueous humor TAC and AA levels in patients with IECD to compare their respective values with those from a control group. This allowed us to then analyze and report on the relationship between aqueous humor TAC/AA and CEC health.

## 2 RESULTS

### 2.1 Study population

A total of 173 subjects were enrolled and examined between April 2019 and March 2020. All patients underwent ocular examinations and aqueous humor aspiration without any adverse events. Aqueous humor TAC and AA were measured in 164 samples and antioxidant proteomics were performed in nine samples, respectively. Using existing medical records, ECD was evaluated in 129 patients. Of these patients, six demonstrated ECD <2100 cells/mm^2^ and were categorized into the IECD group, and 123 displayed ECD >2100 cells/mm^2^ and were categorized into the control group. Supporting Information Figure S1 is a subject recruitment flow diagram. In the IECD group, five patients were diagnosed with cataracts and one was diagnosed with bullous keratopathy. In the control group, 121 patients had cataracts, one had macular degeneration, and one was diagnosed with retinal detachment. The basic characteristics of each group of patients are summarized in Table 1. No meaningful differences in demographics and clinical characteristics were observed (*p* value from 0.105 to 1.000).

**TABLE 1:** Patient characteristics

	All patients	Patient with ECD < 2100 (cells/mm^2^)	Patient with ECD > 2100 (cells/mm^2^)	
Basic characteristics	(N = 164)	(N = 6)	(N = 123)	*p*‐Value
OD/OS, n	73/91	5/1	57/66	0.105^α^
Age, mean ± SD (years)	66.90 ± 10.81	70.67 ± 8.19	65.95 ± 10.29	0.196^β^
Gender, M/F	89/75	5/1	61/62	0.208^β^
BMI, mean ± SD	25.01 ± 3.61	23.22 ± 1.46	25.17 ± 3.68	0.242^β^
Disease diagnosis, n	Cataract: 157/others: 7^a^	Cataract: 5/others: 1^a^	Cataract: 121/others: 2	0.134^α^
Underlying disease				
Hypertension, n (%)	75 (46%)	1 (17%)	60 (49%)	0.212^α^
Diabetes mellitus, n (%)	42 (26%)	1 (17%)	25 (20%)	1^α^
Other underlying systemic diseases, n (%)	74 (45%)	1 (17%)	59 (48%)	0.215^α^

Abbreviations: α, Fisher's exact test; β, Mann–Whitney *U* test; OD, oculus dextrus; OS, oculus sinister; BMI, body mass index; M, male; F, female.

Other disease diagnosis including bullous keratopathy (one patient), macular degeneration (three patients), and retinal detachment (three patients).

### 2.2 CuTAC assay

**FIGURE 2:**
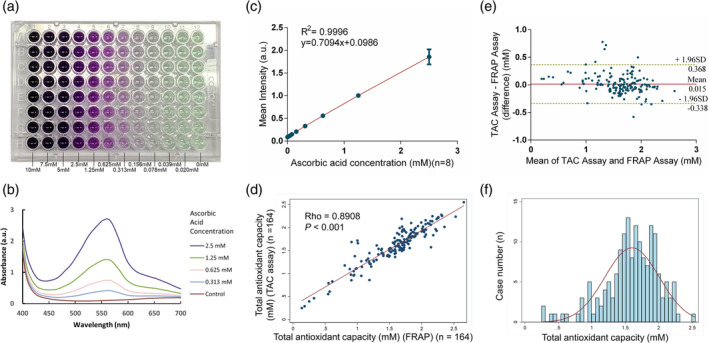
Cupric ion‐based total antioxidant capacity (CuTAC) assay performance. (a) Digital image showing the colorimetric results of the CuTAC assay with serial diluted ascorbic acid (AA) concentrations ranging 0.020–10 mM. The violet color was formed by the chelating reactions between the reduced Cu^1+^ and bicinchoninic acid. (b) The absorption spectra of the CuTAC assay under visible region from 400 to 700 nm. One peak was observed at around 570 nm with the intensity proportional to AA concentration. (c) The calibration curve (*R* ^2^ = 0.9996) of the CuTAC assay under 570 nm with limit of detection (LOD) of 0.016 mM; limit of quantitation (LOQ) of 0.053 mM; and intraassay coefficient of variation (CV) of 4.25% (n = 8). (d) Scatter plot showing a high correlation between the CuTAC and FRAP assay with a Spearman correlation coefficient of 0.891 (*p* value <0.001). (e) Bland–Altman plot showing that there was no significant concentration‐dependent bias between the CuTAC and FRAP assay, with a mean difference of 0.015 mM AA equivalent antioxidant capacity (AAEAC) (95% confidence interval (CI) between 0.368 and −0.338 AAEAC). (f) Histogram showing the distribution of TAC in human aqueous humor (n = 164). It is a left‐skewed distribution with a mean of 1.603; highest level of 2.55; and lowest level of 0.25 mM AAEAC

To meet the clinical needs of aqueous humor analysis, we developed a colorimetric CuTAC assay with an extremely tiny‐sample‐volume requirement, that is, 10 μL per test. We examined colorimetric assay color performance for different AA concentrations as demonstrated in Figure 2a. The resulting violet color was produced by the interaction of bicinchoninic acid (BCA) and Cu^1+^, which is reduced from Cu^2+^ by antioxidants. Higher antioxidant concentrations produced more intensely violet color results. The absorption spectra of the CuTAC assay under the visible light region are illustrated in Figure 2b, where one peak was observed at ~570 nm. Based on these findings, we measured the colorimetric results at 570 nm to yield quantitative information for all subsequent experiments. Tests performed using standard solutions demonstrated an approximately linear relationship between CuTAC assay color intensity and AA concentration ranging from 0.020 to 2.5 mM (*R* ^2^ = 0.9996) (Figure 2c). The calculated limit of detection (LOD) was 0.016 mM; the limit of quantitation (LOQ) was 0.053 mM; the intraassay coefficient of variability (CV) was 4.25% (n = 8); and the interassay CV was 4.13% (n = 19). Verification of the CuTAC assay was performed using a commercially available ferric reducing antioxidant power (FRAP) assay, which demonstrated a high level of consistency and agreement. For verification studies, the Spearman correlation coefficient was 0.891 (n = 164, *p* value <0.001) (Figure 2d). A Bland–Altman analysis demonstrated that the mean difference between the two assays was 0.015 mM AA equivalent antioxidant capacity (AAEAC), with a 95% confidence interval (CI) between 0.368 and −0.338 AAEAC. There was no significant concentration‐dependent bias between the two assays (Figure 2e). We have further tested the stability of the CuTAC assay under different pH conditions, and found that it produced consistent results under a wide range of pH levels from pH 4 to pH 10 (Supporting Information Figure S2). When testing the stability of CuTAC assay at room temperature, the CuTAC assay also exhibited consistent results over time, from 0 to 210 min (Supporting Information Figure S3). Based on these results, we concluded that the CuTAC assay demonstrated good performance and high reproducibility. Moreover, it was as accurate as the commercially available FRAP assay and showed superior tolerability to different pH condition than the FRAP assay, which requires an acidic pH (3.6) to maintain iron solubility. For these reasons, we feel that the CuTAC assay could be an adequate biochemical analytical tool for aqueous humor TAC analysis because the average aqueous humor pH is 7.4 in normal condition.

After evaluating and establishing CuTAC assay performance values, we used it to measure TAC in 164 aqueous humor samples. The mean TAC value of samples tested was 1.603 ± 0.406 mM AAEAC. Figure 2f illustrates the distribution (left‐skewed).

### 2.3 AA concentration and antioxidant proteins in aqueous humor

**FIGURE 3:**
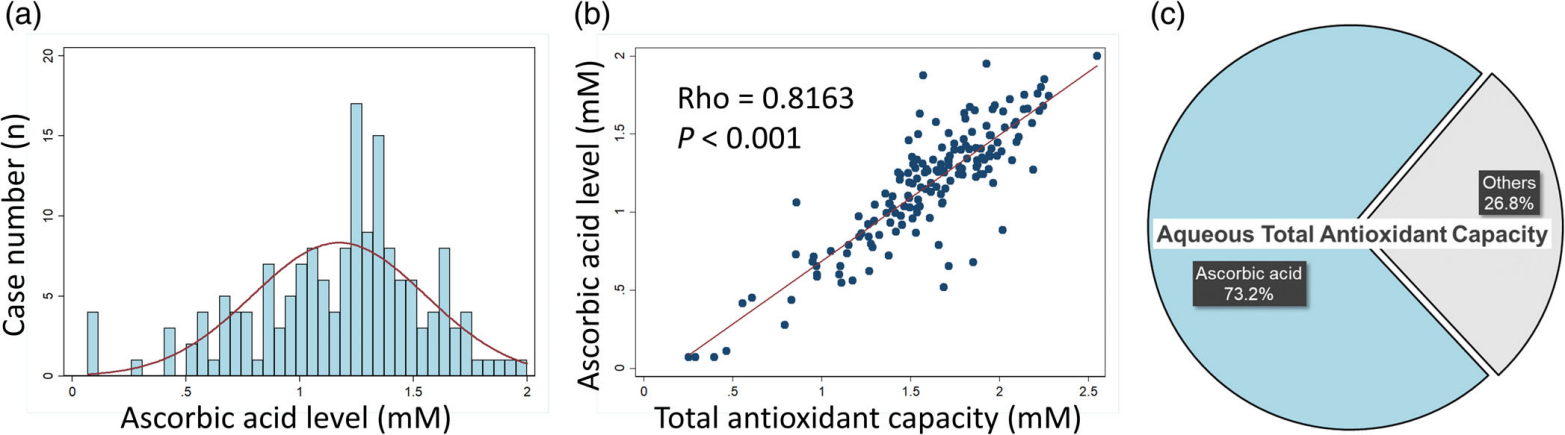
Ascorbic acid (AA) concentration in human aqueous humor and its correlation to aqueous humor total antioxidant capacity (TAC). (a) Histogram showing the distribution of AA in human aqueous humor (n = 164). It is a left‐skewed distribution with a mean of 1.173; highest level of 2; and lowest level of 0.068 mM. (b) Scatter plot showing a high correlation between aqueous humor AA and TAC level with a Spearman correlation coefficient of 0.816 (*p* value <0.001). (c) Pie chart showing the proportional correlation between aqueous humor AA concentration and TAC. The AA averagely contributed to 73.2% of the aqueous humor TAC in human samples

We also measured AA concentration in 164 aqueous humor samples and discovered a left‐skewed distribution (Figure 3a). The mean AA value of all samples tested was 1.173 ± 0.381 mM. We found a high correlation between aqueous humor TAC and AA concentration, with a Spearman correlation coefficient of 0.816 (n = 164, *p* value <0.001) (Figure 3b). Moreover, AA was found to be the primary antioxidant component in aqueous humor, as it was accountable for up to 73.2% of aqueous humor TAC (Figure 3c).

**FIGURE 4:**
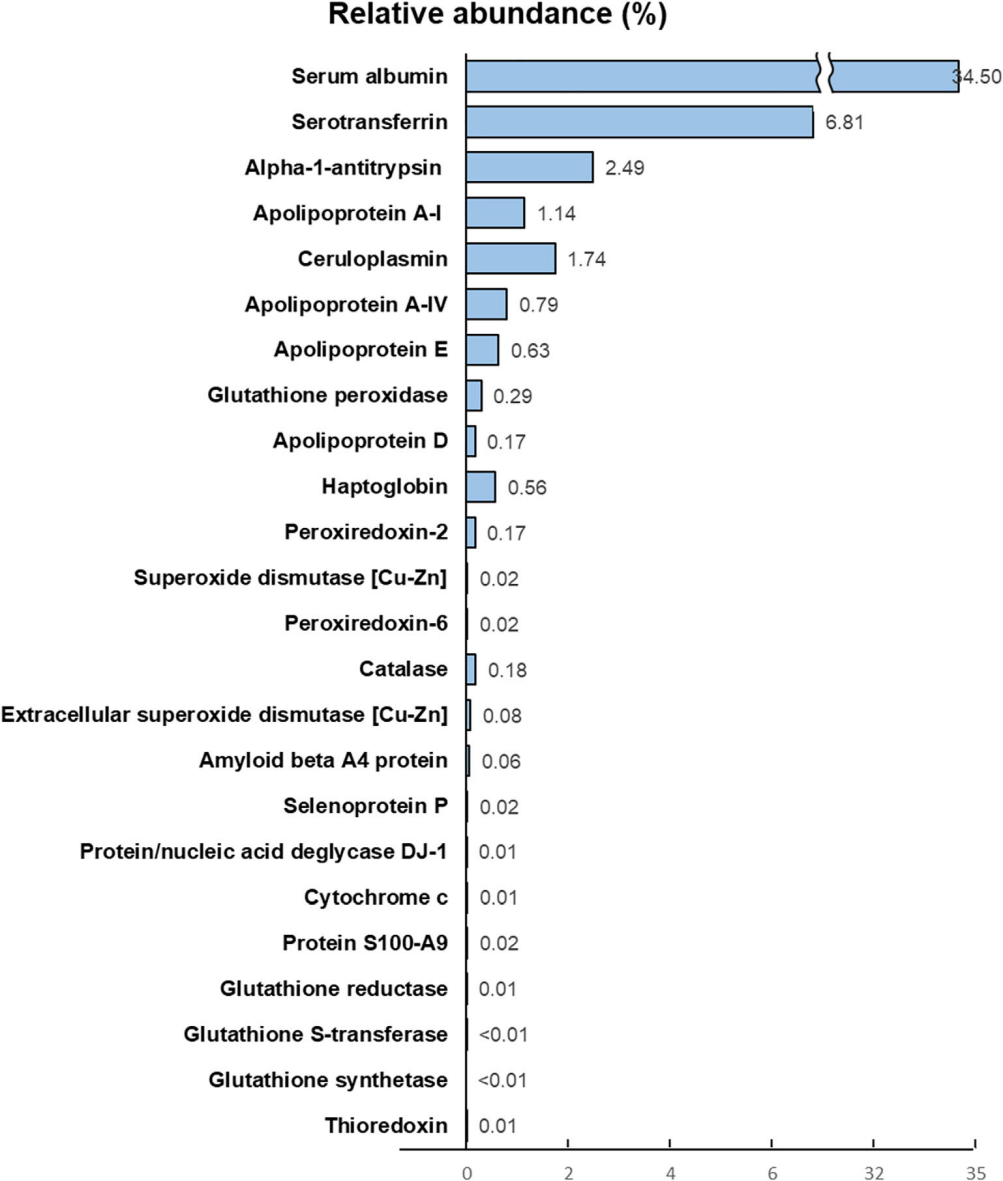
Relative abundance of the identified antioxidant proteins in human aqueous humor. Among the 357 different kinds of protein revealed in aqueous humor, a total of 24 types of antioxidant proteins were identified. Their average relative abundance was calculated as a proportion of specific peptide‐spectrum matches (PSM) within the total PSM (n = 9). Serum albumin, serotransferrin, alpha‐1‐antitrypsin, ceruloplasmin, and apolipoprotein A‐I were found, in that order, to be the most abundant

Regarding proteomics, LC–MS/MS analysis was performed on nine aqueous humor samples. A total of 357 different kinds of protein were found. Among them, 24 kinds of antioxidant proteins were identified; their basic information is listed in Table S1. Most of them demonstrated antioxidative ability to reduce oxidants, especially H_2_O_2_, while others acted as metal‐binding antioxidants, lipoprotein‐related antioxidants, or auxiliary agents of other antioxidants.^23^, ^24^, ^25^, ^26^, ^27^, ^28^, ^29^, ^30^ Functional information for the identified antioxidant proteins is summarized in Table S2. The relative abundance of all identified antioxidant proteins is illustrated in Figure 4, with the top five antioxidant proteins, that is, serum albumin, serotransferrin, alpha‐1‐antitrypsin, ceruloplasmin, and apolipoprotein A‐I, in sequence.

### 2.4 The influence of aqueous humor TAC and AA concentration on CEC health and ocular biometric characteristics

**FIGURE 5:**
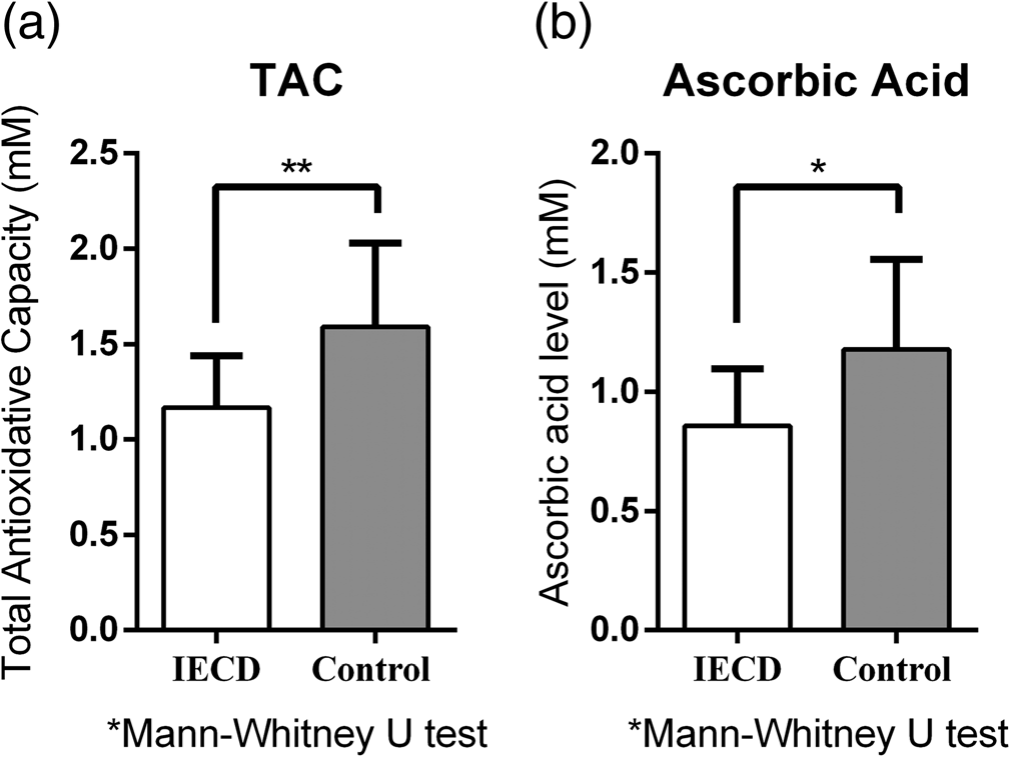
Comparison of aqueous humor total antioxidant capacity (TAC) and ascorbic acid (AA) level between patients with or without insufficient endothelial cell density (IECD). (a) The mean level of aqueous humor TAC was 1.168 ± 0.271 mM AA equivalent antioxidant capacity (AAEAC) in patients with IECD, and 1.592 ± 0.437 mM AAEAC in the control group. The difference was significant when analyzed by the Mann–Whitney *U* test (*p* = 0.009). (b) The mean level of aqueous humor AA was 0.856 ± 0.241 mM in patients with IECD and 1.178 ± 0.376 mM in the control group. The difference was significant when analyzed by the Mann–Whitney *U* test (*p* = 0.016)

The corresponding aqueous humor TAC and AA value of all the samples with different ECD values are illustrated in Supporting Information Figure S4. We then compared the TAC and AA values from the IECD and control group samples. In the IECD group, the mean TAC was 1.168 ± 0.271 mM AAEAC and the mean AA concentration was 0.856 ± 0.241 mM. In the control group, the mean TAC was 1.592 ± 0.437 mM AAEAC, and the mean AA concentration was 1.178 ± 0.376 mM. There were significant differences between both TAC and AA levels between groups (*p* = 0.009 for TAC difference and *p* = 0.016 for AA difference) (Figure 5a,b).

To further evaluate the influence of aqueous humor TAC and AA concentration on CEC health, we examined the effects of both on ECD. We used logistic regression to obtain crude and adjusted odds ratios. The crude odds ratio for the influence of TAC and AA concentration on IECD were 0.184 with a *p* value of 0.029, and 0.149 with a *p* value of 0.049, respectively. To determine the adjusted odds ratio, all of the parameters mentioned in Table 1 including age, sampling eye site, gender, body mass index, ocular diseases, and underlying systemic diseases were considered as confounding factors and adjusted by using multivariate logistic regression analysis. For aqueous humor TAC, the adjusted odds ratio was 0.02 with 95% CI between 0.001 and 0.499 (*p* value = 0.017) (i.e., every 1 mM of elevated TAC in the aqueous humor would reduce the risk for developing IECD by 50 times); for aqueous humor AA, the adjusted odds ratio was 0.023 with a 95% CI between 0.001 and 0.736 (*p* value = 0.033) (i.e., every 1 mM of elevated AA in the aqueous humor would reduce the risk for developing IECD by 43 times) (Table 2). These results indicate that TAC and AA concentration are independent protective factors against IECD.

**TABLE 2:** Association of total antioxidative capacity and ascorbic acid level in aqueous humor with endothelial cell density

	Crude odds ratio		Adjusted odds ratio	
	OR (95% CI)	*p*‐Value	OR (95% CI)	*p*‐Value
TAC (mM)	0.184 (0.04–0.843)	0.029^a^	0.02 (0.001–0.499)	0.017^a^
Ascorbic acid level (mM)	0.149 (0.022–0.991)	0.049^a^	0.023(0.001–0.736)	0.033^a^

Abbreviations: CI, confidence interval; OR, odds ratio; TAC, total antioxidant capacity.

a Age, eye site, gender, body mass index, disease diagnosis, and underlying diseases were adjusted as the confounding factors.

We also examined other endothelial characteristics, including CV, hexagonal cell ratio (HEX), and central corneal thickness (CCT), and compared the results obtained from both IECD and control group patients. We found that CV value was higher in the IECD group than in the control group, but there were no significant differences in other endothelial characteristics between groups (Supporting Information Figure S5A–C). We also found no significant differences between groups for ophthalmic structural parameters, including axial length (AL), anterior chamber depth (ACD), cylinder correction (cyl), and spherical equivalent (SE) (Supporting Information Figure S5D–H). The analyzed *p* values are provided in Supporting Information Figure S5I.

## 3 DISCUSSION

In this study, we developed a colorimetric CuTAC assay that required only a 10 μL sample per test to provide a complete analysis. Although several kinds of spectrophotometric assays for TAC measurement have been developed, such as the oxygen radical absorbance capacity (ORAC) assay, the DPPH assay, the trolox equivalent antioxidant capacity (TEAC) assay, and the ferric reducing antioxidant power (FRAP) assay, our CuTAC assay was rapid, inexpensive, easy to use, highly accurate, and only required a small sample volume.^31^, ^32^ In addition, the CuTAC assay demonstrated stable performance under a wide range of pH levels and time spans at room temperature. It could, therefore, be an ideal platform for small‐volume TAC detection and relevant clinical applications, that is, disease evaluation and treatment efficacy assessment. For clinical application, we measured the TAC level in 164 aqueous humor samples and completed all analyses in triplicate without diluting the samples, which may have a negative impact on accuracy.

We extended our investigation to examine the major small molecular and protein‐based antioxidants within aqueous humor. Interestingly, we found that AA contributed up to 73.2% of aqueous humor TAC. This is a comparatively high proportion when you consider that uric acid, the most potent free radical scavenger in serum, contributes only up to 50% of serum TAC.^33^, ^34^ However, our results are in agreement with others showing that AA modulated the overall antioxidative effects of aqueous humor.^35^, ^36^ AA in aqueous humor was reported to act as an ultraviolet filter, a direct free radical scavenger, and an antioxidant regulator.^37^, ^38^ Furthermore, AA can counteract the induction of apoptosis and lipid‐peroxidation.^9^ This study has built upon previous research and is the first to simultaneously detect aqueous humor TAC and AA, and further quantify the proportion of AA to TAC. Although preventive AA supplementation was suggested to reduce endothelial cell loss during phacoemulsification,^18^, ^19^ it should be used with caution because high concentrations of AA may have adverse effects on CEC.^39^ This study provided evidence to support pretreatment evaluation of TAC and AA level, but more comprehensive clinical trials examining the possible benefit of AA supplementation are warranted.

In some regards, the antioxidant proteins identified in aqueous humor showed a certain degree of similarity with those of serum. In both systems, albumin appears to be the most abundant antioxidant protein, and metal‐binding proteins such as serotransferrin and ceruloplasmin also play important roles in both antioxidant defense systems.^40^, ^41^ Additionally, it has been reported that aqueous humor AA concentration positively and directly correlates with H_2_O_2_ level.^42^ This may be due to the antioxidant capacity of AA, which can transform the superoxide anion into H_2_O_2_. This finding fit our proteomic results showing that most of the identified antioxidant proteins exhibit their primary antioxidant ability in eliminating H_2_O_2_ (Table S2). Based on these findings, we believe that AA and antioxidant proteins collaborate with each other to build up the unique antioxidant defense system in aqueous humor. However, further studies on the interaction of aqueous humor antioxidants are required.

To evaluate the influence of aqueous humor TAC and AA concentration on corneal health, we compared aqueous humor TAC and AA concentration in patients with IECD to the same values in a control group and found that both were significantly lower in patients with IECD. Logistic regression results further verified that aqueous humor TAC and AA were actually protective factors against IECD. To the best of our knowledge, this is the first clinical study to report on the positive impacts of aqueous humor TAC and AA on CEC health. Although the exact mechanism underlying this protective effect has not yet been elucidated, several in vitro and animal studies have suggested a relationship between free radical scavengers and reduced CEC damage.^43^, ^44^ Cestmir et al. and Guo‐Jian et al. reported that antioxidants play an important role in the survival, healing, and migration of CEC.^45^, ^46^ This might explain the significant differences in ECD and CV between the two patient groups in our study. Moreover, Tsutomu et al. reported on the results of a double‐blind clinical trial indicating that the use of dissolved H_2_ in irrigation solution reduced corneal endothelial damage during phacoemulsification. This study supports the significant role played by aqueous humor TAC in preventing CEC damage.^47^

In terms of the relationship between aqueous humor TAC and ocular integrity, we found a statistically significant difference in CV levels between the two groups, suggesting reduced CEC uniformity in patients with lower aqueous humor TAC. This result might indicate a relationship between aqueous humor TAC and corneal endothelial CV values. However, the exact correlation requires further exploration. Notably, this study did not reveal any correlation between TAC and AL, ACD, cyl, SE, HEX, or CCT. These results differ slightly from those of Sakae et al., who found reduced aqueous humor AA concentrations in women with smaller ACD.^48^ This discrepancy might be due to the relatively small sample size of both studies. Additional research is required to fully investigate and define the relationship between aqueous humor TAC and ocular biometric characteristics.

While notable for its findings, our study was limited. As a retrospective study, we only measured aqueous humor TAC and AA level once for each patient. This eliminated our capacity to examine fluctuations in aqueous humor TAC and AA concentrations and examine progressive ECD changes. However, previous studies have found ECD to be an important corneal parameter that should be evaluated before ocular surgery.^6^, ^7^, ^8^ Our novel insight into the association between aqueous humor TAC and IECD supports the importance of aqueous humor TAC. Consequently, evaluation of aqueous humor TAC and AA concentration can be considered a promising route for developing biochemical analysis designed to guide ophthalmological treatment. Another significant limitation of our study was the small number of IECD patients enrolled. Despite rigorously adjusting the confounding factors to reduce possible bias, further large‐scale clinical trials are required to verify our findings.

This study had several limitations. Because it required an invasive procedure to obtain the aqueous humor sample, we were not allowed to recruit healthy subjects for the control group. It was also difficult to monitor the aqueous humor TAC level with repeated, longitudinal follow‐up settings. Another significant limitation of our study was the small number of IECD patients enrolled. To overcome these limitations and provide a trustworthy result, we designed the study in a rigorous way. We analyzed all the samples in triplicate to prevent experimental bias. In addition, we adjusted all potential confounding factors in the regression models to reduce selection bias. However, further large‐scale clinical trials are required to verify our findings.

In conclusion, we developed a CuTAC assay to accurately measure aqueous humor TAC without the need of sample dilution. We also analyzed aqueous humor AA concentration and antioxidant proteomics and found that AA was accountable for the majority of aqueous humor TAC. In the case–control portion of our study, we found that both aqueous humor TAC and AA level were significant protective factors against IECD. These results demonstrate the positive influence of TAC and AA on corneal health and suggest the need for further studies to evaluate the treatment efficacy of AA supplementation to mitigate ECD loss.

## 4 MATERIALS AND METHODS

### 4.1 Patients

This is a case–control study approved by the institutional review board (IRB) of Chang Gung Memorial Hospital in 2019 (IRB number: 201900017B0) and carried out in accordance with all the relevant guidelines and tenets of the Declaration of Helsinki. All patients recruited in this study provided written informed consent before enrollment. Eligible patients were aged >18 years who underwent aqueous humor sample collection before their scheduled ophthalmological operation from April 1, 2019 to March 31, 2020 in Chang Gung Memorial Hospital. Exclusion criteria included glaucoma, diabetic retinopathy, infectious ocular diseases, shallow anterior chamber, branch retinal vein occlusion, pterygium, Sjogren's disease, corneal transplant status, and postoperation status. These underlying ocular diseases and conditions were considered to either influence TAC or interfere with ECD measurement.^11^, ^12^, ^13^, ^14^, ^15^ A flow diagram for subject recruitment is presented in Supporting Information Figure S1. In brief, among the 205 patients who received aqueous humor aspiration before eye surgery, 173 were included in this study. From that 173, 164 aqueous humor samples were randomly selected for aqueous humor TAC and AA analysis, and the other nine were selected to participate in the aqueous humor antioxidant proteomics group. In the analysis of aqueous humor TAC and AA group, patients who had their ECD evaluated before the surgery were further classified into the IECD group (ECD < 2100 cells/mm^2^) or the control group (ECD > 2100 cells/mm^2^) according to their baseline ECD value.

### 4.2 Sample collection and clinical data acquisition

Aqueous humor samples were aspirated just before a patient's scheduled ophthalmological operation. At the beginning of the surgery, a 27‐gauge needle was carefully inserted into the anterior chamber through the paralimbal cornea, and 50–100 μL of aqueous humor was obtained. Once the samples were collected, they were immediately stored at −80°C before being analyzed. For data acquisition, all the clinical data were acquired from the electronic medical record. The corneal endothelium was evaluated using a noncontact in vivo specular microscope (CEM‐530, Nidek, Gamagori, Japan), and parameters including ECD, CV of the cell area, and HEX were recorded. Other ocular biometry including CCT, AL, ACD, cyl, and SE were measured using an autorefractometer (KR‐7000, Tokyo, Japan) and IOLMaster (Carl Zeiss Meditec, Inc., Dublin, CA).

### 4.3 Development of total antioxidant capacity analytical assay

Assays for TAC measurement can be classified into direct and indirect types. Direct type TAC assays measure the ability of target samples to eliminate free radicals and inhibit oxidation.^31^ Indirect TAC assays evaluate the ability of target sample to reduce a metal ion. In this study, we developed an indirect type, small‐sample‐volume, colorimetric TAC assay based on cupper (II) redox reactions, that is, a CuTAC assay. Reagents including BCA and CuSO_4_ were purchased from Thermo Fisher Scientific (contained in the Pierce™ BCA Protein Assay Kit, Catalog number: 23225, Waltham, MA, USA). The AA, used for the standard solution preparation, was purchased from Fisher Scientific (B581‐05, JT Baker, Phillipsburg, USA).

For the analytical process, 10 μL of serial diluted AA solution ranging from 0.02 to 2.5 mM as the calibration curve and undiluted aqueous humor sample were added into 96‐well microplates in triplicate. We then added 200 μL of 0.08% CuSO_4_ solution diluted with BCA and incubated for 20 min away from light. During the incubation period, antioxidant compounds in the solution reduced the Cu^2+^ to Cu^1+^, and Cu^1+^ interacted with BCA to form a violet chelate complex. Colorimetric results were measured at 570 nm using an absorbance microplate reader (Sunrise™ Tecan, Switzerland). Because we used AA to establish our calibration curve, the measured TAC is expressed as AAEAC.

We used a commercially available FRAP assay to verify the accuracy and utility of the CuTAC assay.^49^ We also examined CuTAC assay stability under varying pH conditions, from pH 4 to pH 10, and over different time spans at room temperature, from 0 to 210 min, before any analytical processes.

### 4.4 AA measurement and antioxidant proteomics

Aqueous humor AA concentration was measured using a colorimetric OxiSelect™ Ascorbic Acid Assay Kit (FRASC, Cell Biolabs, Inc., San Diego, CA, USA).^50^ Samples were diluted 20 times in assay buffer and analyzed in triplicate.

To examine antioxidant proteomics, we used the in‐sol digestion method to prepare the samples. In brief, 50 mM ammonium bicarbonate (Sigma‐Aldrich, USA) was used to dilute the samples, and 10 mM dithiothreitol (Merck, Darmstadt, Germany) was added for the reduction reaction. For cysteine‐blocking, samples were incubated with 40 mM iodoacetamide (Sigma, St. Louis, MO, USA) at 25°C for 30 min. Finally, the sequencing‐grade modified porcine trypsin (Promega, Madison, WI, USA) was used to digest the sample at 37°C for 16 h. The digested peptides were then desalted, dried, and store at −80°C until analysis.

We used liquid chromatography based tandem‐mass spectrometry (LC–MS/MS) to identify and quantify the compounds in aqueous humor. The 1D‐LC (RP), Dionex Ultimate 3000 RSLCnano system (Thermo Fisher Scientific, Waltham, MA, USA) was used in conjunction with a hybrid mass spectrometer, the thermo Orbitrap Elite (Thermo Fisher Scientific, Waltham, MA, USA). A full‐scan MS scan was carried out in the Orbitrap at a resolution of 120,000 at m/z 400, followed by an MS/MS scan for the 20 most abundant precursor ions appearing in the MS. All data were analyzed using Proteome Discoverer software (version 2.2, Thermo Fisher Scientific). In addition, we searched the SwissProt database (extracted for *Homo sapiens*) for the MS/MS spectra interpretation using the Mascot search engine (Matrix Science, London, UK; version 2.5). Peptide‐spectrum matches (PSM) were filtered using the high‐confidence and Mascot search engine, and relative abundance (%) as the semiquantitative scale for the discovered protein was defined as the proportion of the specific PSM in the total PSM.^51^ Finally, results were compared with The Antioxidant Protein Database (http://lin-group.cn/AODdatabase/Browse.aspx) for antioxidant protein identification. The functional information of the identified proteins was obtained using the UniProt Knowledgebase (UniProtKB, https://www.uniprot.org/) and relevant journal articles.^52^, ^53^

### 4.5 Statistical analysis

We performed statistical analyses using Stata software version 14 (StataCorp LP, College Station, TX, USA). A *p* value of less than 0.05 was considered statistically significant. Descriptive statistics were used for patient characterizations, which were presented as means with SDs or proportions as appropriate. For continuous data, we tested the normality of the distribution using the Kolmogorov–Smirnov test. The linearity of the calibration curve of the CuTAC assay was expressed through the coefficient of determination (*R* ^2^). The limit of detection (LOD) and limit of quantitation (LOQ) were calculated by the following equations

(1) $$\mathrm{LOD}=\frac{3\times \mathrm{SD}\;\text{of the bank}}{\text{slope}}$$

(2) $$\mathrm{LOQ}=\frac{10\times \mathrm{SD}\;\text{of the bank}}{\text{slope}}$$

The intraassay CV and interassay CV of the CuTAC assay were calculated using the ratio between the SD and mean intensity of the blank value. Spearman correlation and Bland Altman analyses were used to analyze the relationship and agreement between the CuTAC assay and the FRAP assay. Aqueous humor TAC and AA concentration distribution were demonstrated in a histogram. The proportional correlation of aqueous humor AA in TAC was presented as a pie chart and analyzed using the Spearman correlation analysis. To compare values between IECD and control groups, we used the Student's *t* test for the normally‐distributed data, and the Mann–Whitney *U* test for the non‐normally distributed data. We further used univariate and multivariate logistic regression analyses to calculate crude and adjusted odds ratios to examine the influence of aqueous humor TAC and AA concentration on ECD. In the multivariate logistic regression analysis, age, sampling eye site, gender, body mass index, ocular diseases, and systemic diseases were considered confounding factors and adjusted.

## AUTHOR CONTRIBUTIONS

**Yu‐Ting Tsao:** Conceptualization; data curation; formal analysis; investigation; methodology; project administration; software; visualization; writing‐original draft. **Wei‐Chi Wu:** Conceptualization; data curation; investigation; project administration; resources. **Kuan‐Jen Chen:** Conceptualization; data curation; project administration; resources. **Lung‐Kun Yeh:** Formal analysis; resources; validation. **Yih‐Shiou Hwang:** Resources; validation. **Yi‐Jen Hsueh:** Formal analysis; investigation; project administration; validation. **Hung‐Chi Chen:** Conceptualization; data curation; investigation; methodology; project administration; resources; supervision; writing‐review and editing. **Chao‐Min Cheng:** Conceptualization; data curation; funding acquisition; investigation; methodology; project administration; supervision; writing‐review and editing.

## CONFLICT OF INTEREST

The authors have no conflicts of interest to declare.

### PEER REVIEW

The peer review history for this article is available at https://publons.com/publon/10.1002/btm2.10199.

## Supporting information


**Figure S1** Flow diagram presenting the recruitment process of the study subjects.

**Figure S2** The performance of cupric ion‐based total antioxidant capacity (CuTAC) assay under different pH level from pH 4 to pH 10. The CuTAC assay showed highly linear relationship with serial ascorbic acid concentrations under different pH level. The overlapping line charts was illustrated at the bottom of the figure

**Figure S3** The performance of cupric ion‐based total antioxidant capacity (CuTAC) assay under different starting time point in room temperature. The CuTAC assay showed highly linear relationship with serial ascorbic acid concentrations under different starting time point in room temperature. The overlapping line charts was illustrated at the bottom of the figure.

**Figure S4** The correlation between total antioxidant capacity (TAC), ascorbic acid (AA), and endothelial cell density (ECD). (A) Scatter plot showing no significant linear relationship between aqueous humor TAC and ECD value. However, the patients with IECD (red spots) showed relatively lower TAC than the control group patients (navy spots). If the possible outliers were deleted, the difference in aqueous humor TAC between patients with IECD and control group patients would be more significant. (B) Scatter plot showing no significant linear relationship between aqueous humor AA concentration and ECD value. However, the patients with IECD (red spots) showed relatively lower AA values than the control group patients (navy spots). If the possible outliers were deleted, the difference in aqueous humor AA between patients with IECD and control group patients would be more significant.

**Figure S5** The comparison of corneal endothelial and biometrical parameters between patients with insufficient endothelial cell density (IECD) and the control group.

**Table S1** List of antioxidant proteins identified in the aqueous humor proteomics.

**Table S2** Functions of the antioxidant proteins identified in the aqueous humor proteomics.

## Data Availability

The data that support the findings of this study are available from the corresponding author upon reasonable request.
