# Supplementary material for: Analysis of aqueous humor total antioxidant capacity and its correlation with corneal endothelial health
Source: Bioeng Transl Med. 2020 Dec 5;6(2):e10199. doi: 10.1002/btm2.10199 (PMC8126826; doi:10.1002/btm2.10199)
Supplement: Supplementary file 1 — Figure S1 Flow diagram presenting the recruitment process of the study subjects. [file BTM2-6-e10199-s004.pdf]

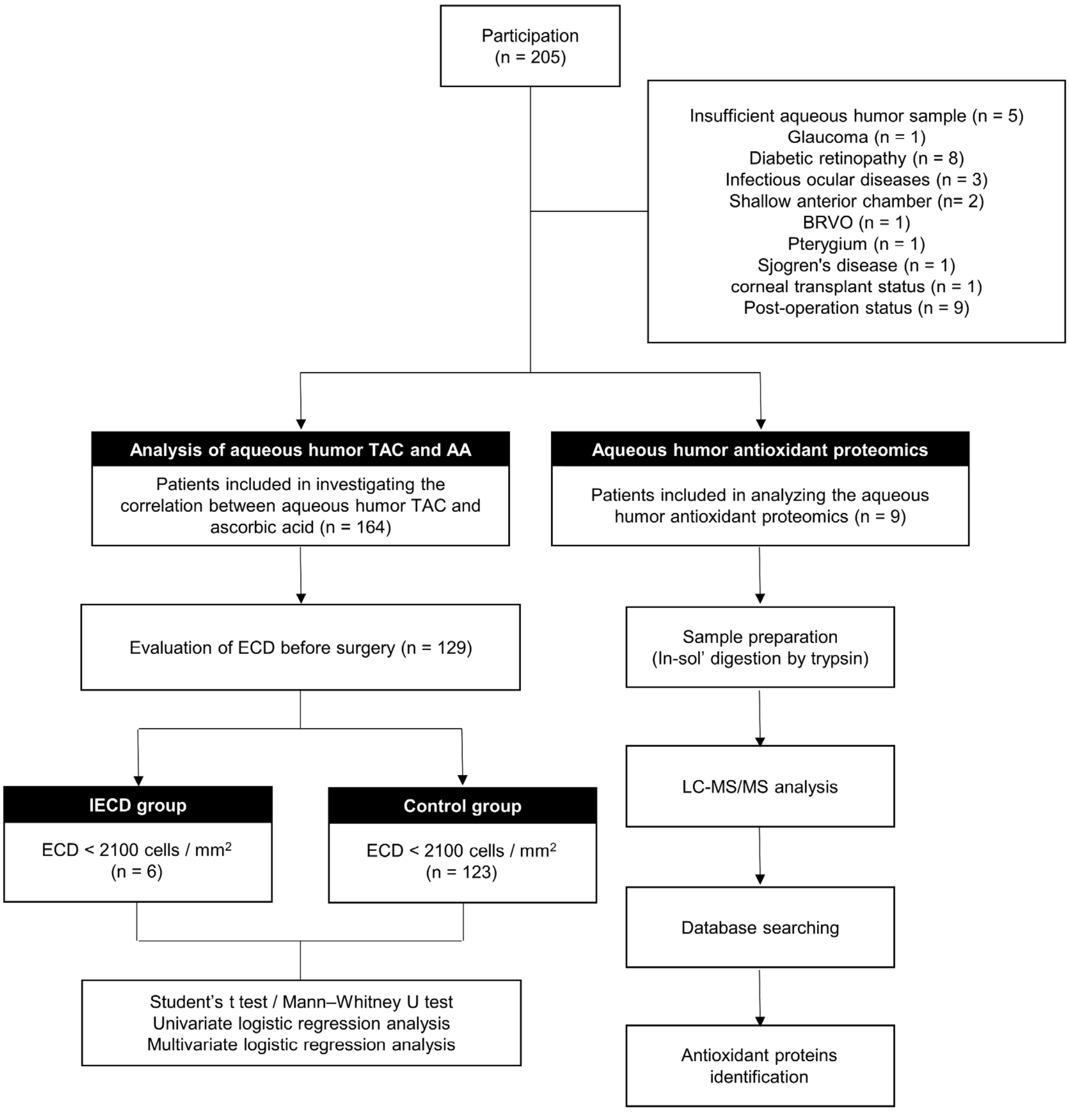

AA = ascorbic acid; BRVO = branch retinal vein occlusion; ECD = endothelial cell density; IECD = insufficient endothelial cell density; TAC = total antioxidant capacity; LC-MS/MS = Liquid chromatography-tandem mass spectrometry

Supplementary Figure 1. Flow diagram presenting the recruitment process of the study subjects.
