# Supplementary material for: Analysis of aqueous humor total antioxidant capacity and its correlation with corneal endothelial health
Source: Bioeng Transl Med. 2020 Dec 5;6(2):e10199. doi: 10.1002/btm2.10199 (PMC8126826; doi:10.1002/btm2.10199)
Supplement: Supplementary file 3 — Figure S3 The performance of cupric ion‐based total antioxidant capacity (CuTAC) assay under different starting time point in room temperature. The CuTAC assay showed highly linear relationship with serial ascorbic acid concentrations under different starting time point in room temperature. The overlapping line charts was illustrated at the bottom of the figure. [file BTM2-6-e10199-s001.pdf]

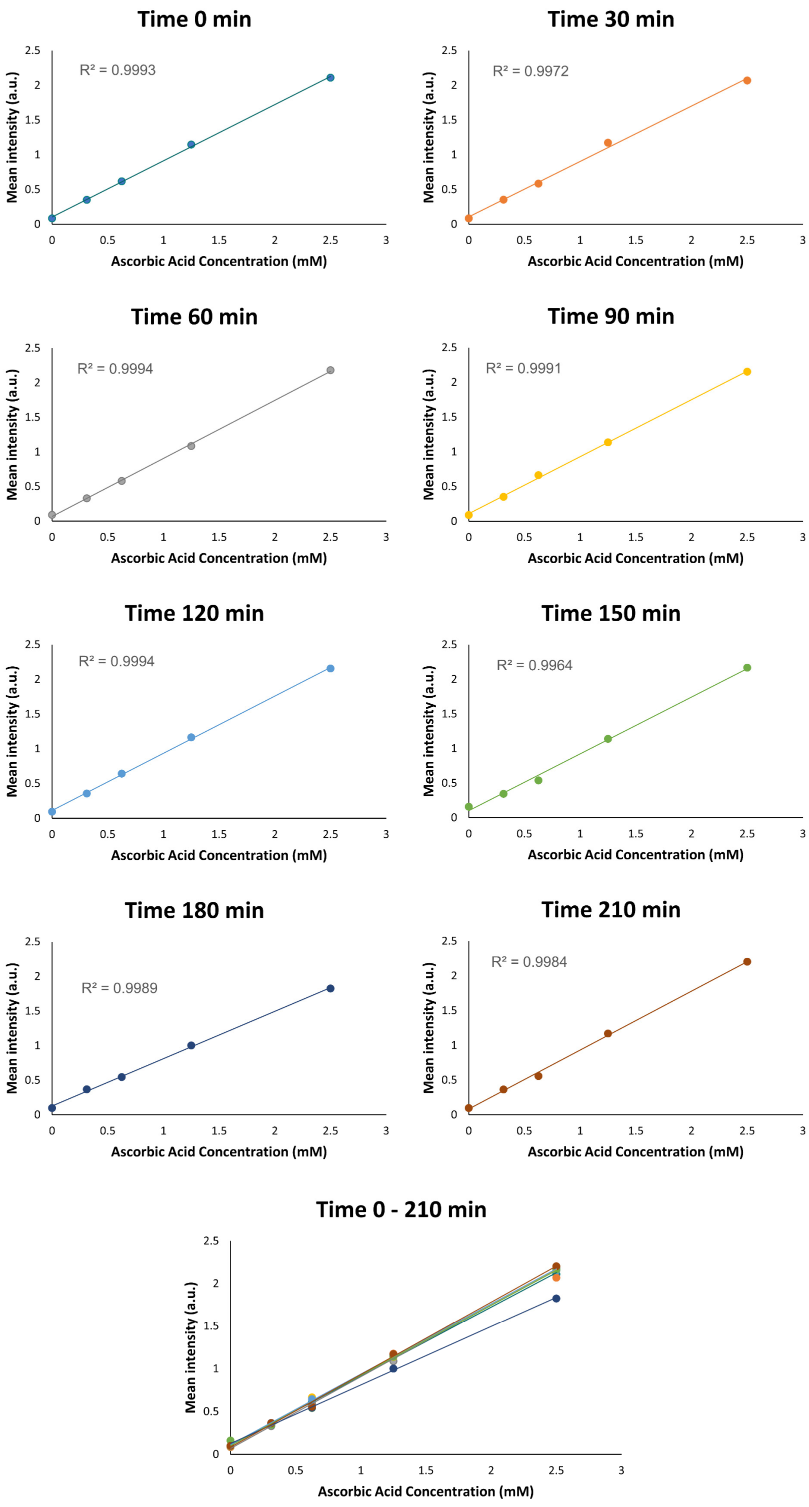

Supplementary Figure 3. The performance of cupric ion-based total antioxidant capacity (CuTAC) assay under different starting time point in room temperature. The CuTAC assay showed highly linear relationship with serial ascorbic acid concentrations under different starting time point in room temperature. The overlapping line charts was illustrated at the bottom of the figure.
