# Supplementary material for: Analysis of aqueous humor total antioxidant capacity and its correlation with corneal endothelial health
Source: Bioeng Transl Med. 2020 Dec 5;6(2):e10199. doi: 10.1002/btm2.10199 (PMC8126826; doi:10.1002/btm2.10199)
Supplement: Supplementary file 4 — Figure S4 The correlation between total antioxidant capacity (TAC), ascorbic acid (AA), and endothelial cell density (ECD). (A) Scatter plot showing no significant linear relationship between aqueous humor TAC and ECD value. However, the patients with IECD (red spots) showed relatively lower TAC than the control group patients (navy spots). If the possible outliers were deleted, the difference in aqueous humor TAC between patients with IECD and control group patients would be more significant. (B) Scatter plot showing no significant linear relationship between aqueous humor AA concentration and ECD value. However, the patients with IECD (red spots) showed relatively lower AA values than the control group patients (navy spots). If the possible outliers were deleted, the difference in aqueous humor AA between patients with IECD and control group patients would be more significant. [file BTM2-6-e10199-s006.pdf]

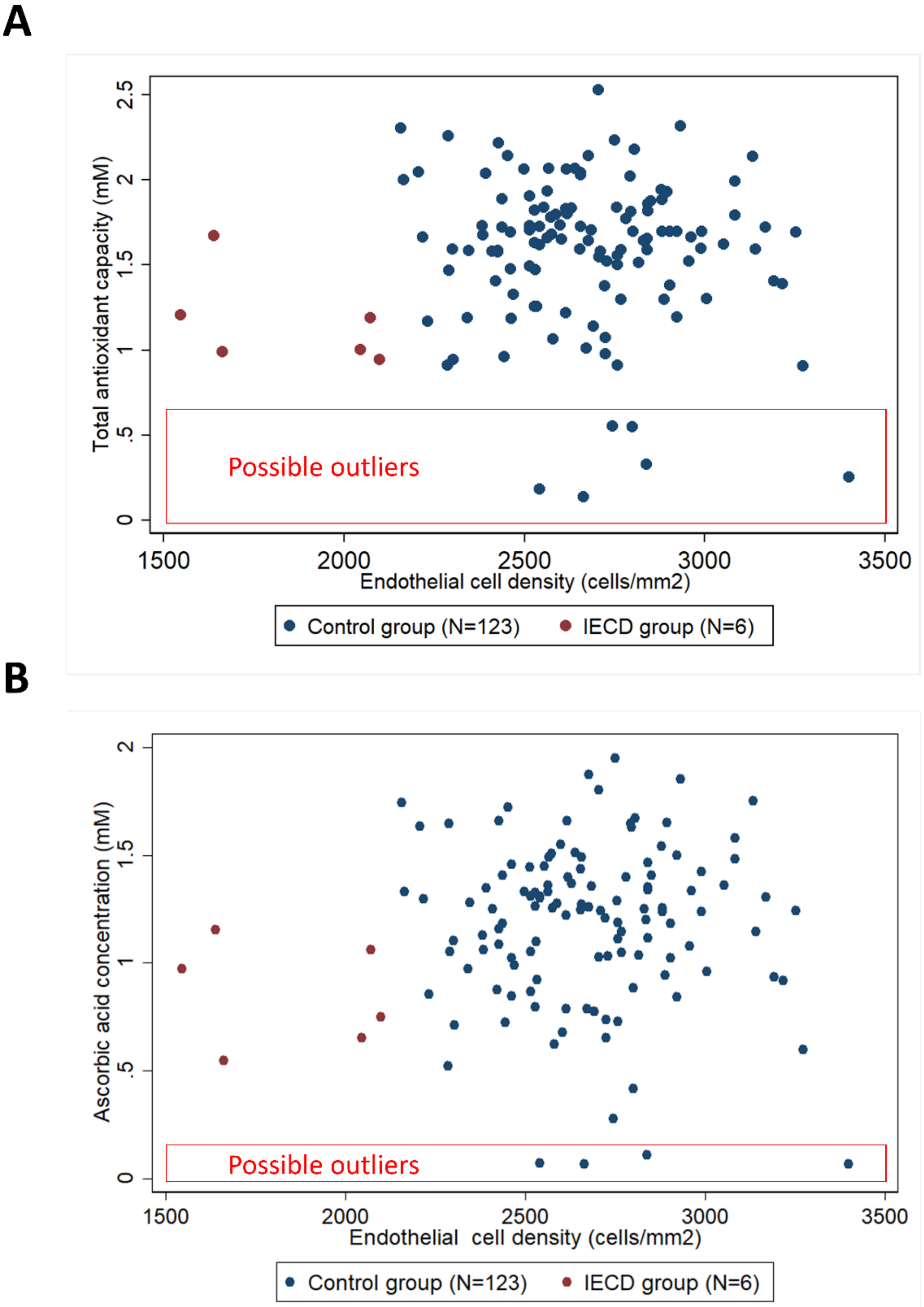

Supplementary Figure 4. The correlation between total antioxidant capacity (TAC), ascorbic acid (AA), and endothelial cell density (ECD). (A) Scatter plot showing no significant linear relationship between aqueous humor TAC and ECD value. However, the patients with IECD (red spots) showed relatively lower TAC than the control group patients (navy spots). If the possible outliers were deleted, the difference in aqueous humor TAC between patients with IECD and control group patients would be more significant. (B) Scatter plot showing no significant linear relationship between aqueous humor AA concentration and ECD value. However, the patients with IECD (red spots) showed relatively lower AA values than the control group patients (navy spots). If the possible outliers were deleted, the difference in aqueous humor AA between patients with IECD and control group patients would be more significant.
