# Supplementary material for: Analysis of aqueous humor total antioxidant capacity and its correlation with corneal endothelial health
Source: Bioeng Transl Med. 2020 Dec 5;6(2):e10199. doi: 10.1002/btm2.10199 (PMC8126826; doi:10.1002/btm2.10199)
Supplement: Supplementary file 5 — Figure S5 The comparison of corneal endothelial and biometrical parameters between patients with insufficient endothelial cell density (IECD) and the control group. [file BTM2-6-e10199-s002.pdf]

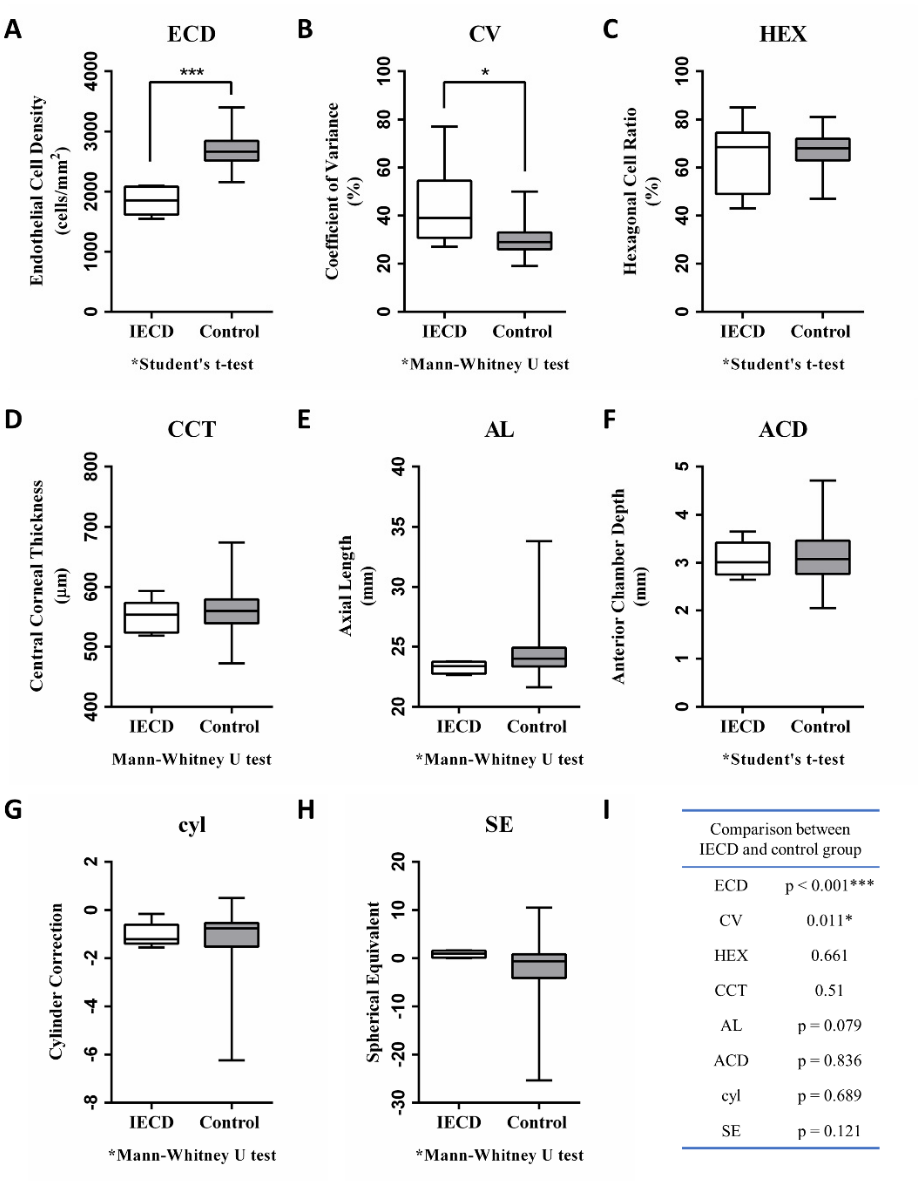

Comparison between IECD and control group	
ECD	p < 0.001***
CV	0.011*
HEX	0.661
CCT	0.51
AL	p = 0.079
ACD	p = 0.836
cyl	p = 0.689
SE	p = 0.121

Supplementary Figure 4. The comparison of corneal endothelial and biometrical parameters between patients with insufficient endothelial cell density (IECD) and the control group. (A) The endothelial cell density (ECD) value was significantly lower in the IECD group compared to the control group. (1843.83 ± 253.08 vs. 2677.68 ± 252.42 cells / mm2, $P < 0.001$) (B) The coefficient of variation (CV) value was significantly higher in the IECD group compared to the control group. (43.5 ± 17.87 vs. 29.9 ± 5.88 %, $P = 0.011$) (C) There was no significant difference between both groups in hexagonal cell ratio (HEX) value. (64.5 ± 15.1 vs. 67.38 ± 6.21 %, $P = 0.661$) (D) There was no significant difference between both groups in central corneal thickness (CCT) value. (551.33 ± 27.69 vs. 560.07 ± 33.84 µm, $P = 0.51$) (E) There was no significant difference between both groups in axial length (AL) value. (23.3 ± 0.52 vs. 24.66 ± 2.26 mm, $P = 0.079$) (F) There was no significant difference between both groups in anterior chamber depth (ACD) value. (3.07 ± 0.38 vs. 3.12 ± 0.47 mm, $P = 0.836$) (G) There was no significant difference between both groups in cylinder correction (cyl) value. (-1.04 ± 0.53 vs. -1.07 ± 0.91, $P = 0.689$) (H) There was no significant difference between both groups in spherical equivalent (SE) value. (0.88± 0.76 vs. -2.77 ± 6.91 mm, $P = 0.121$) (I) The analyzed *P* value of above-mentioned parameters. There were only significant differences in ECD and CV values between IECD and control group. All of these comparisons were analyzed by Student's t test or Mann-Whitney U test.
