# Supplementary material for: Analysis of aqueous humor total antioxidant capacity and its correlation with corneal endothelial health
Source: Bioeng Transl Med. 2020 Dec 5;6(2):e10199. doi: 10.1002/btm2.10199 (PMC8126826; doi:10.1002/btm2.10199)
Supplement: Supplementary file 6 — Table S1 List of antioxidant proteins identified in the aqueous humor proteomics. [file BTM2-6-e10199-s003.pdf]

Table S1. List of antioxidant proteins identified in the aqueous humor proteomics

Protein name	Accession	Peptides	Unique Peptides	Coverage (%)	MudPIT score	MW [kDa]	PSM
Serum albumin	P02768	31	31	58.95	1324.87	69.32	1277.5
Serotransferrin	P02787	33	33	60.6	2179.02	77.01	253
Alpha-1-antitrypsin	P01009	10	10	28.23	548.87	46.71	91.25
Apolipoprotein A-I	P02647	10	10	43.07	645.27	30.76	43.25
Ceruloplasmin	P00450	40	40	49.2	3888.08	122.13	63.75
Apolipoprotein A-IV	P06727	18	18	51.26	694.84	45.37	30.25
Apolipoprotein E	P02649	9	9	37.85	518.80	36.13	23.5
Glutathione peroxidase	P22352	6	6	32.3	661.29	25.54	6.25
Apolipoprotein D	P05090	4	4	25.93	122.47	21.26	10.75
Haptoglobin	P00738	2	2	8.37	54.03	45.18	22.5
Peroxiredoxin-2	P32119	7	7	28.79	237.02	21.88	6.75
Superoxide dismutase [Cu-Zn]	P00441	5	5	60.39	249.60	15.93	0.75
Peroxiredoxin-6	P30041	2	2	9.38	37.23	25.02	0.75
Catalase	P04040	11	11	27.51	581.25	59.72	6.75
Extracellular superoxide dismutase [Cu-Zn]	P08294	7	7	39.58	497.49	25.83	3.6
Amyloid beta A4 protein	P05067	7	7	13.12	357.51	86.89	2.6
Selenoprotein P	P49908	2	2	4.72	141.77	43.16	0.8
Protein/nucleic acid deglycase DJ-1	Q99497	2	2	17.46	63.43	6.79	0.4
Cytochrome c	P99999	2	2	29.52	63.11	11.74	0.6
Protein S100-A9	P06702	2	2	17.54	53.01	13.23	1
Glutathione reductase	P00390	1	1	2.49	37.29	56.22	0.4
Glutathione S-transferase	P09211	1	1	4.76	36.81	23.34	0.2
Glutathione synthetase	P48637	1	1	1.69	24.13	52.35	0.2
Thioredoxin	P10599	1	1	12.38	136.89	11.73	0.4

Peptides: number of identified peptides to a protein group; Unique peptides: number of unique peptides (unshared peptides) to the protein; Coverage: peptide sequence coverage of identified protein (%); MudPIT score: sum of the excess of ions score of each peptide; MW [kDa]: Molecular weight of identified protein ; PSM: Peptide-spectra matches
