# Supplementary material for: Analysis of aqueous humor total antioxidant capacity and its correlation with corneal endothelial health
Source: Bioeng Transl Med. 2020 Dec 5;6(2):e10199. doi: 10.1002/btm2.10199 (PMC8126826; doi:10.1002/btm2.10199)
Supplement: Supplementary file 7 — Table S2 Functions of the antioxidant proteins identified in the aqueous humor proteomics. [file BTM2-6-e10199-s007.pdf]

Table S2. Functions of the antioxidant proteins identified in the aqueous humor proteomics[1]

Protein name	Antioxidative activity	Other functions
**Elimination of oxidants**		
Serum albumin	Free radical-trapping[2]	Regulation of the colloidal osmotic pressure<br>Transportation of zinc, calcium, and magnesium
Glutathione peroxidase	Catalyzation of the reduction of H2O2, lipid peroxides and organic hydroperoxide	
Peroxiredoxin-2	Catalyzation of the reduction of H_2O_2 and organic hydroperoxides<br>Regulation of the intracellular H_2O_2 concentration	
Superoxide dismutase [Cu-Zn]	Catalyzation of the reduction of $^{\bullet}O2^{-}$	
Peroxiredoxin-6	Catalyzation of the reduction of H_2O_2 and organic hydroperoxides<br>Protection of phospholipid from oxidative damage	
Catalase	Catalyzation of the reduction of H_2O_2	Promotion of cell growth
Extracellular superoxide dismutase [Cu-Zn]	Catalyzation of the reduction of $^{\bullet}O2^{-}$	
Protein/nucleic acid deglycase DJ-1	Oxidative stress sensor<br>Elimination of H_2O_2<br>Reduction of copper<br>Interaction with NADPH oxidase	Neuroprotection
Cytochrome c	Inhibition of H_2O_2 production<br>Catalyzation of the reduction of $^{\bullet}O2^{-}$	Electron carrier<br>Inducement of apoptosis
Protein S100-A9	Oxidant scavenger	Modulation of the inflammatory and immune response
**Metal-binding protein**		
Serotransferrin	Iron-binding antioxidant capacity[3]	Stimulation of cell proliferation
Ceruloplasmin	Iron-binding antioxidant capacity[3]<br>Auxiliary of ascorbic acid	Development of fetal lung
Amyloid beta A4 protein	Reduction of copper<br>Protection of lipoprotein from metal-catalyzed oxidation	Promotion of neuronal growth, adhesion and axonogenesis

**Lipoprotein-related antioxidant protein**		
Apolipoprotein A-I	Protection of phospholipid from oxidative damage[4]	Transportation of cholesterol to the liver
Apolipoprotein A-IV	Inhibition of lipoprotein oxidation[5]	Secretion and catabolism of chylomicrons and VLDL<br>Transportation of cholesterol to the liver
Apolipoprotein D	Protection of lipid from oxidative damage<br>Reduction of hydroperoxyeicosatetraenoic acid[6]	Transportation and binding of bilin
Apolipoprotein E	Protection of cells from beta-amyloid peptides[7]	Transportation of lipid between organs<br>Production, conversion and clearance of lipoprotein
**Auxiliary of other antioxidants**		
Selenoprotein P	Auxiliary of selenium	
Glutathione reductase	Reduction of glutathione<br>Catalyzation of the reduction of $NADP^+$	
Glutathione S-transferase	Conjugation of reduced glutathione	Prevention of neurodegeneration
Glutathione synthetase	Synthesis of glutathione	
**Others**		
Thioredoxin	Catalyzation of the dithiol-disulfide exchange reactions	
Alpha-1-antitrypsin	Inhibition of insulin-induced NO synthesis<br>Protection of lung from smoking injury[8]	Inhibition of proteases including trypsin, chymotrypsin, plasminogen etc.
Haptoglobin	Protection of cells from hemoglobin-driven oxidative damage[9]	Recycle of heme iron<br>Antibacterial activity<br>Modulation of the acute phase response

NADPH: Nicotinamide adenine dinucleotide phosphate; $NADP^+$: glyoxylate reductase; VLDL: very low-density lipoproteins

REFERENCE

1. Consortium TU. UniProt: a worldwide hub of protein knowledge. *Nucleic Acids Research*. 2018;47(D1):D506-D515. doi:10.1093/nar/gky1049

2. Roche M, Rondeau P, Singh NR, Tarnus E, Bourdon E. The antioxidant properties of serum albumin. *FEBS letters*. 2008;582(13):1783-1787.

3. Golizeh M, Lee K, Ilchenko S, et al. Increased serotransferrin and ceruloplasmin

turnover in diet-controlled patients with type 2 diabetes. *Free Radical Biology and Medicine*. 2017;113:461-469.
4. Bielicki JK, Oda MN. Apolipoprotein A-IMilano and apolipoprotein A-IParis exhibit an antioxidant activity distinct from that of wild-type apolipoprotein AI. *Biochemistry*. 2002;41(6):2089-2096.
5. Qu J, Ko C-W, Tso P, Bhargava A. Apolipoprotein A-IV: A Multifunctional Protein Involved in Protection against Atherosclerosis and Diabetes. *Cells*. 2019;8(4):319.
6. Bhatia S, Knoch B, Wong J, et al. Selective reduction of hydroperoxyeicosatetraenoic acids to their hydroxy derivatives by apolipoprotein D: implications for lipid antioxidant activity and Alzheimer's disease. *Biochemical Journal*. 2012;442(3):713-721.
7. Miyata M, Smith JD. Apolipoprotein E allele–specific antioxidant activity and effects on cytotoxicity by oxidative insults and β–amyloid peptides. *Nature genetics*. 1996;14(1):55-61.
8. Marzatico F, Gaetani P, Tartara F, et al. Antioxidant status and α1-antiproteinase activity in subarachnoid hemorrhage patients. *Life sciences*. 1998;63(10):821-826.
9. Melamed-Frank M, Lache O, Enav BI, et al. Structure-function analysis of the antioxidant properties of haptoglobin. *Blood, The Journal of the American Society of Hematology*. 2001;98(13):3693-3698.
